# The Role of Myeloid-Derived Cells in the Progression of Liver Disease

**DOI:** 10.3389/fimmu.2019.00893

**Published:** 2019-04-24

**Authors:** Chris John Weston, Henning Wolfgang Zimmermann, David H. Adams

**Affiliations:** ^1^Centre for Liver and Gastrointestinal Research, Institute of Immunology and Immunotherapy, Medical School, University of Birmingham, Birmingham, United Kingdom; ^2^NIHR Birmingham Biomedical Research Centre, University Hospitals Birmingham NHS Foundation Trust and University of Birmingham, Birmingham, United Kingdom; ^3^Medical Department III, University Hospital of Aachen, Aachen, Germany

**Keywords:** hepatitis (general), hepatocellular carcinoma, cirrhosis, fibrosis, myeloid derived suppressor cell (MDSC), neutrophil (PMN), macrophage, circulating monocytes

## Abstract

Control of homeostasis and rapid response to tissue damage in the liver is orchestrated by crosstalk between resident and infiltrating inflammatory cells. A crucial role for myeloid cells during hepatic injury and repair has emerged where resident Kupffer cells, circulating monocytes, macrophages, dendritic cells and neutrophils control local tissue inflammation and regenerative function to maintain tissue architecture. Studies in humans and rodents have revealed a heterogeneous population of myeloid cells that respond to the local environment by either promoting regeneration or driving the inflammatory processes that can lead to hepatitis, fibrogenesis, and the development of cirrhosis and malignancy. Such plasticity of myeloid cell responses presents unique challenges for therapeutic intervention strategies and a greater understanding of the underlying mechanisms is needed. Here we review the role of myeloid cells in the establishment and progression of liver disease and highlight key pathways that have become the focus for current and future therapeutic strategies.

## Introduction

Myeloid cells arise from the common myeloid precursor and give rise to monocytes, dendritic cells and macrophages, and granulocytes. Myeloid cell functions include the recognition, ingestion and degradation of cellular debris, foreign material or pathogens, subsequent control of inflammatory responses, and maintenance of tissue architecture. There is increasing evidence implicating granulocytes in liver homeostasis and disease but this review will focus mainly on monocytes and macrophages. Macrophages are a diverse, heterogeneous population derived from short-lived, but plastic, precursor monocyte populations. Monocytes are rapidly recruited to sites of injury and their functions are imprinted in the bone marrow, whereas macrophages tend to be long-lived and tissue-resident, where their functions are dictated by environmental cues (1). A highly coordinated pathway of monocyte recruitment and subsequent imprinting of macrophage “identities,” the mechanisms of which are only now beginning to be understood, controls local tissue inflammatory and regenerative functions and is critical in maintaining tissue architecture (2–4). Extensive rodent and human studies have demonstrated key roles for monocytes and macrophages in the establishment, progression and regression of liver disease including a critical role in directing tissue regeneration (5–7). This fine balance of pro- and anti-inflammatory mediators is crucial to determining the path of disease progression, and understanding how myeloid cells contribute to injury and repair will enable the rational design of novel therapies. In this review we summarize the identities and roles of myeloid cell populations in the liver, and describe approaches that are being developed to reduce inflammation through targeting this innate immune cell population.

## Hepatic Monocyte and Macrophage Populations

### Kupffer Cells

**Figure 1:**
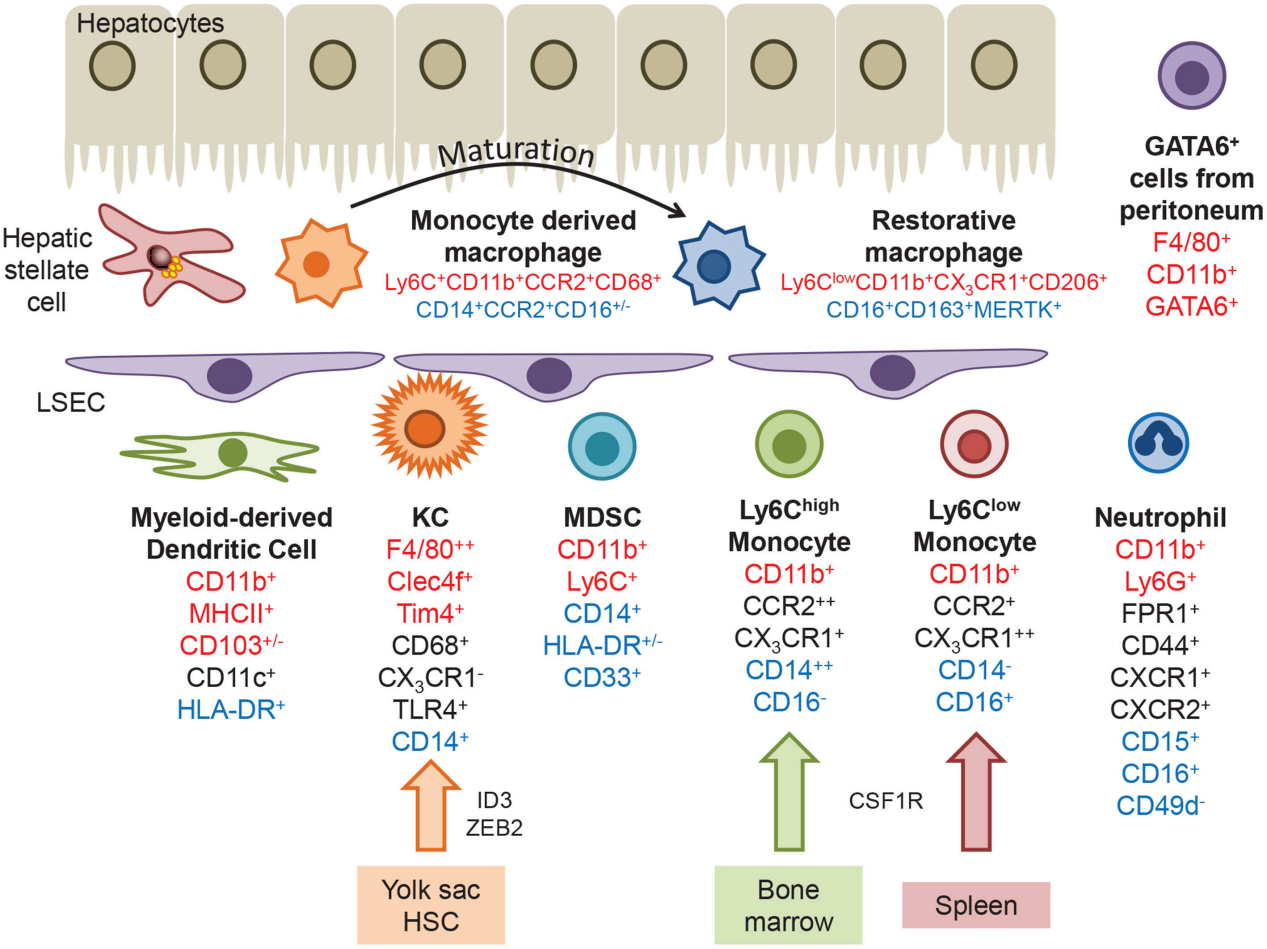
Myeloid populations present within the liver. Kupffer cells are derived from the yolk sac or hematopoietic stem cells, under the control of transcription factors such as ID3 and ZEB2. Infiltrating monocytes originating in the bone marrow or spleen express the chemokine receptors CCR2 and CX_3_CR1, and can differentiate into monocyte-derived dendritic cells. Following injury monocytes undergo transendothelial migration across LSEC and differentiate into monocyte derived macrophages, which can mature into a more restorative phenotype or replenish the KC pool. A GATA6^+^ macrophage population that migrates from the peritoneum during hepatic injury has been identified in mice. Markers that identify hepatic macrophages in mice, humans or are common to both are highlighted in red, blue and black text, respectively.

The liver contains a population of self-renewing resident macrophages, termed Kupffer cells (KC), derived from yolk sac-derived progenitor cells (8, 9) or hematopoietic stem cells (10). In mice KC phenotype is thought to be controlled by a specific set of transcription factors including ID3 and ZEB2 through progenitor cell development and maintenance of the expression of LXRα permitting replenishment of the KC niche by progenitors from the circulation (2, 11–13) (Figure 1, Table 1). They are non-migratory, being retained in the sinusoids where they maintain a tolerogenic environment despite the presence of low levels of food particles and bacterial antigens delivered from the gut via the portal vein (4, 14, 15). This is achieved through highly effective phagocytic and scavenging mechanisms triggered by toll-like receptor (TLR) signaling and scavenger receptors such as CD36, scavenger receptor-A and galectin-3 (16, 17). Their expression of high levels of pattern recognition receptors (PRRs) allows macrophages to respond to a wide range of danger-associated molecular patterns (DAMPs) released during tissue injury, such as high mobility group protein B1 (HMGB1), ATP, uric acid, DNA fragments and cholesterol crystals (5) and pathogen-associated molecular patterns (PAMPs, such as lipopolysaccharide and flagellin) released from microbes. Activation of PRRs leads to the formation of the inflammasome (18) via multi-protein complexes including the NOD-, LRR- and pyrin domain-containing 3 (NLRP3) (19). Formation of the inflammasome promotes the release of potent signaling molecules including IL-1β, PGE_2_, HMGB1, TNF-α, and IL-17, driving inflammation and fibrosis (20–24). KC from both mouse and human liver also secrete the anti-inflammatory cytokine IL-10 (25–27), express low levels of MHC class II and co-stimulatory molecules combined with high levels of the T-cell inhibitory molecule PDL-1 (27). This makes them unable to fully activate T cell effector function but rather to promote the development of regulatory T-cells (Treg). This is further enhanced through their secretion of PGE_2_ (28) and upregulation of the indolamine 2,3-dioxygenase pathway which promotes immune cell tolerance (29).

**Table 1:** Myeloid cell populations in humans and mice.

**Myeloid population**	**Murine markers**	**Human markers**	**Role**
Myeloid-derived dendritic cell	CD11b^+^ MHCII^+^ CD11c^+^ CD103^+/−^	CD11c^+^ HLA-DR^+^	Tolerogenic in nature; Upon injury may adopt an inflammatory phenotype; Functional role in liver disease ill-defined
Kupffer cells	CD11b^+^ CD68^+^ F4/80^++^ CLEC4f^+^ TIM4^+^ CX_3_CR1^−^ TLR4^+^ TLR9^+^ CRIg^+^	CD68^+^ CD14^+^ TLR4^+^ CX_3_ CR1^−^	Promote tolerance under steady-state conditions to restrict immune response against food-borne antigens; Activated during tissue damage; main source of cytokines / chemokines governing local inflammation
Myeloid derived suppressor cells	CD11b^+^ Ly6C^+^	CD14^+^ HLA-DR^+/−^ CD33^+^	Immunosuppressive; Facilitate HCC growth by dampening T-cell activity
Monocyte derived macrophage	CD11b^+^ Ly6C^+/−^ F4/80^+/−^ CCR2^+^ CX_3_CR1^+^ CD64^+^	CD14^+^ CCR2^+^ CD16^+/−^	*See subsets below*
Inflammatory macrophage	Ly6C^high^ CD11b^+^ CCR2^++^ CX_3_CR1^+^ iNOS^+^ TNF^+^	CD14^++^ CD16^−^ CLEC5A^+^ S100A9^+^	Pro-inflammatory, massively recruited during liver injury; elicits tissue damage; drive fibrogenesis by maintaining inflammation and activating fibrosis effector cells; can undergo phenotypic switch to restorative macrophages
Pro-resolution macrophage	Ly6C^low^ CD11b^+^ CCR2^+^ CX_3_CR1^++^ CD206^+^ MMP9^+^ MMP12^+^	CD14^−^ CD16^+^ CD163^+^ CCR2^+^ CX_3_ CR1^++^ Stabilin-1^+^ (MERTK^+^)	Anti-inflammatory; restorative function in liver fibrosis; promote tissue repair after acute damage; in humans CD16^+^ rather linked to fibrosis progression
Neutrophils	CD11b+ Ly6G+ Fpr1+ CD44^+^ CXCR1^+^ CXCR2^+^	CD15^+^ CD16^+^ CD49d^−^ FPR1^+^ CD44^+^ CXCR1^+^ CXCR2^+^	Ambiguous role in liver injury; functional role likely context-dependent; putatively profibrogenic in steatohepatitis
Peritoneal infiltrating cells	CD11b^+^ F4/80^+^ GATA6^+^	Unknown	Currently not known

Murine KC have been well characterized under homeostatic conditions and in experimental models of hepatic injury, where they express CD11b^+^F4/80^++^CD68^+^CD11c^+/−^CLEC4F^+^TIM4^+^ in addition to TLR4, TLR9, and CRIg, but are negative for the chemokine receptor CX_3_CR1. Recent advances in proteomic analysis has revealed circadian regulation of not only KC numbers in uninjured mouse liver, but also components of the immune response pathway which peak during the daytime, including Tlr4, Myd88, Irak4, and Tak1 (30). Human KC are less well described but can be identified through expression of CD68^+^CD14^+^TLR4^+^ and lack of CX_3_CR1.

### Infiltrating Monocytes

Circulating monocytes are actively recruited to the liver, guided by adhesion molecules and chemokine gradients generated at the sinusoidal endothelial interface (see below). In mice bone marrow derived myeloid cells expressing high levels of Ly6C and CCR2 rapidly infiltrate tissue and are associated with the expression of pattern recognition receptors (PRR) and inflammatory cytokines (CD11b^+^CCR2^+^CX_3_CR1^+^CD43^−^). In contrast Ly6C^low^ monocytes from the spleen express a broad range of scavenger receptors and exhibit a patrolling behavior that may enable the engulfment of apoptotic cells (CD11b^+^CCR2^−^CX_3_CR1^++^CD43^+^) (27, 31–36). In humans there is no discriminatory expression of Ly6C and monocytes are classified according to the expression of CD14 and CD16 giving rise to classical (CD14^++^CD16^−^), intermediate (CD14^+^CD16^+^), and non-classical (CD14^−^CD16^+^) populations. Gene expression profiling of these subsets has determined that CD14^++^CD16^−^ monocytes resemble murine Ly6C^high^ infiltrative cells and CD14^−^CD16^+^ monocytes more closely align with the patrolling Ly6C^low^ population (31). Potent immunomodulatory myeloid derived suppressor cells (MDSC) are also present in both murine and human liver tissue. MDSCs are a heterogeneous population of cells which express markers shared with other immune cell populations (CD11b^+^Ly6C^+^ in mice, CD14^+^HLA-DR^+/−^CD33^+^ in humans), therefore identification is usually confirmed by means of a T-cell suppression assay (37). MDSC suppress immune responses through production of arginase 1 (Arg1), inducible nitric oxide synthase (iNOS) and generation of reactive oxygen species (ROS), or secretion of IL-10 (38).

## Recruitment From the Circulation and Differentiation in Tissue

Damage to tissue results in an upregulation of adhesion molecules on liver sinusoidal endothelium (LSEC) and the secretion of chemokines, cytokines and other bioactive molecules that promote immune cell recruitment [reviewed in (39)]. Circulating CCR2^+^ monocytes are recruited in response to local CCL2, released primarily by hepatic stellate cells (HSC) (40, 41), or through the CCR8/CCL1 and CXCR3/CXCL10 axes (42–44). In humans the migration of CD14^−^CD16^+^ monocytes is promoted through activation of CX_3_CR1 by endothelial CX_3_CL1, a transmembrane chemokine that is expressed at high levels during inflammation (45). Intermediate CD14^+^CD16^+^ monocyte populations are enriched in the diseased liver (46), partly due to their increased propensity when compared with other monocytes to migrate across LSEC. These cells exhibit high phagocytic activity and secrete pro-inflammatory and fibrogenic mediators (47). Bidirectional migration of monocytes affects the local balance of inflammatory and anti-inflammatory cells. Pro-inflammatory CD14^−^CD16^+^ subsets undergo reverse migration from tissue back into the circulation via across LSEC from where they may contribute to systemic inflammatory responses, whereas anti-inflammatory cells remain in the tissue where they suppress T-cells and promote endotoxin tolerance (48). A phenotypic switch in macrophage phenotype is observed during acute liver injury in humans where MAC387 (S100A9) can be used to identify circulation-derived macrophages in contrast to CD68^+^ resident populations (49). Infiltrating monocytes also undergo local intrahepatic differentiation into anti-inflammatory MDSC following injury via contact-dependent mechanisms such as communication with hepatic stellate cells (HSC), or interaction with soluble mediators such as catalase (50, 51).

In mouse models of sterile injury CCR2^+^Ly6C^high^ monocytes form rings to demarcate the extent of injury (14), and subsequently mature into Ly6C^low^ monocytes that promote the resolution of injury and fibrosis (52). These “pro-restorative” macrophages exhibited a phenotype distinct from the classical M1 (pro-inflammatory) or M2 (pro-resolution) dichotomy with increased expression of genes that promote tissue restoration including matrix metalloproteinases (MMPs), growth factors, and phagocytosis-related genes. Murine monocytes can also take unconventional routes into liver tissue. In a model of sterile liver injury GATA6-positive macrophages (CD11b^+^F4/80^+^Gata6^+^) originating in the peritoneal compartment were observed within the hepatic compartment at a very early stage of tissue damage (53). These cells migrate directly across the mesothelium, dependent on adenosine triphosphate and the adhesion molecule CD44. The contribution of these cells to disease pathogenesis is currently unknown.

Thus, local polarization of myeloid cell populations, and recruitment of macrophages from other sites, has important implications in disease pathogenesis where the balance of pro-/anti- inflammatory mediators and fibrogenic responses dictates the course of the disease.

## Response to Acute and Chronic Liver Disease

### Acute Liver Disease (Such as Acetaminophen Overdose, Acute Viral, or Alcoholic Hepatitis)

**Figure 2:**
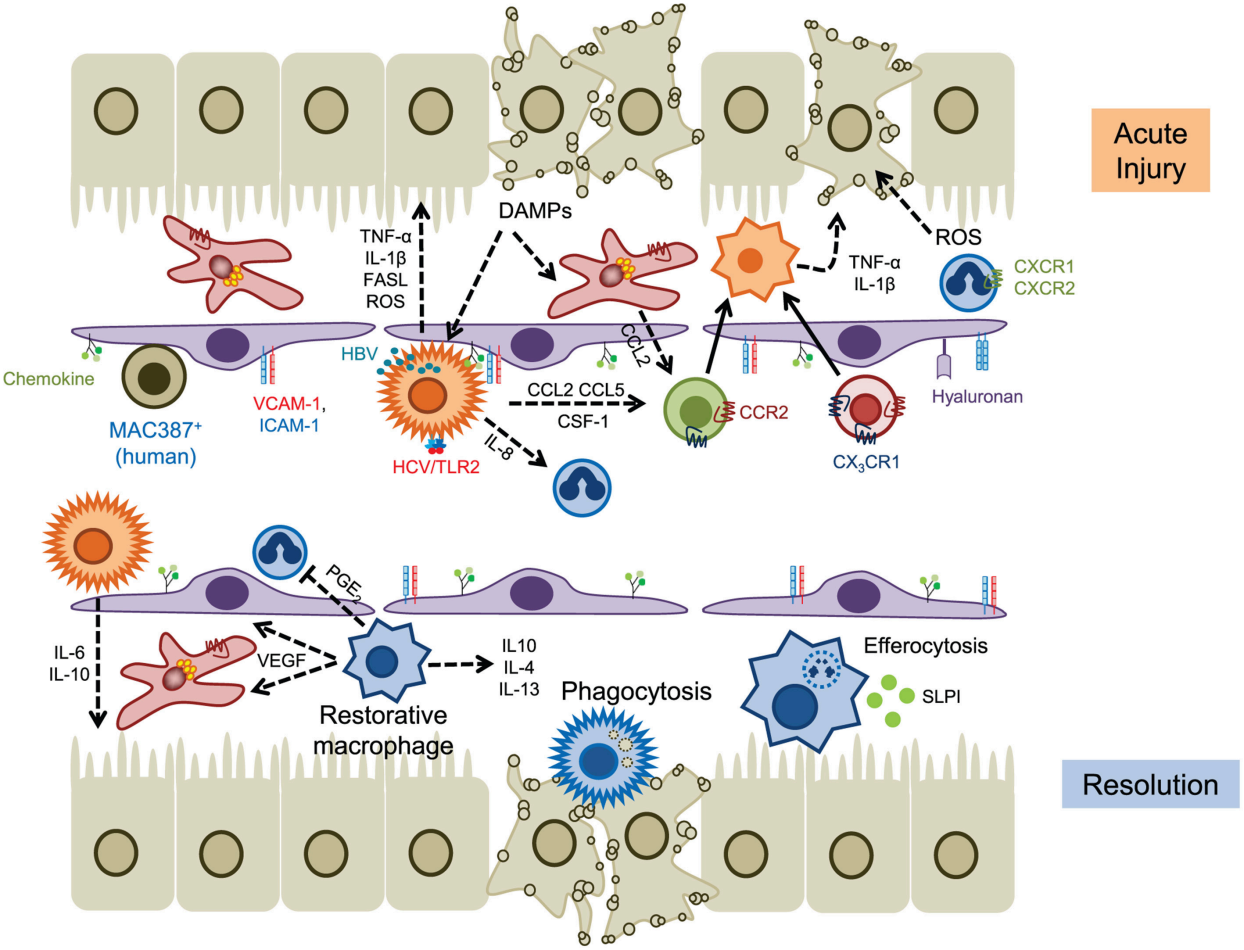
The role of myeloid cells in acute liver injury. Hepatocyte cell death releases DAMPs that activate KC and hepatic stellate cells, leading to the release of chemokines such as CCL2 and IL-8 (CXCL8) that drives the recruitment of myeloid cells into local areas of inflammation (top). Neutrophils are recruited via CD44 and hyaluronan and generate ROS that promotes hepatocyte death, whereas infiltrating monocytes (and KCs) secrete proinflammatory cytokines such as TNFα and IL-1β. Hepatic viruses can also stimulate KC through internalization (HBV) or binding to TLR2 (HCV). Homeostasis is restored through the action of restorative macrophages (matured by phagocytosis) that secrete anti-inflammatory cytokines and promote angiogenesis, and secretion of IL-6 and IL-10 by KC (lower panel). Infiltrating neutrophils are removed through efferocytosis mediated by MERTK^+^ macrophages and SLPI. Solid lines indicate cell migration, dashed lines represent the secretion of soluble mediators.

Acute liver failure (ALF) is associated with high mortality and toxic liver injury in response to overdose of drugs such as acetaminophen is a more common cause of ALF than immune-mediated injury arising from acute viral hepatitis (54, 55). Much of what we know of macrophage function during early disease is derived from experimental models of acute liver injury in rodents such as carbon tetrachloride toxicity (hepatocyte necrosis) (56), bacterial infection (57), concanavalin A (T cell mediated hepatocyte destruction) (58), ischemia-reperfusion (I-R) injury (59), sterile injury (14), and viral infection (60). These models show that extensive hepatocyte damage mediated by heat/toxin/immune-mediated killing releases DAMPs such as HMGB1 and nuclear DNA which are sensed by KC leading to the release of cytokines and chemokines, creating an environment that drives the recruitment of inflammatory macrophage subsets (5). In the absence of persistent injury, tissue repair is initiated by the maturation of pro-inflammatory populations to a more restorative phenotype, associated with anti-inflammatory and pro-angiogenic responses (52) (Figure 2).

In acetaminophen (APAP) induced liver injury, perhaps the best described rodent model of ALF, KC respond to tissue damage through the rapid release of cytokines and chemokines including IL-1β, TNF-α, CCL2, and CCL5 (61). Initially the numbers of KC are reduced (<24 h) and early injury is associated with high numbers of infiltrating Ly6C^high^ monocytes which produce proinflammatory cytokines such as TNF-α and IL-1β and chemokines such as CCL2 and CCL5 (62–65). Evidence that these early entrants drive tissue injury comes from data showing that (i) infiltration of these cells during acetaminophen-induced injury can be reduced through blockade of CCR2-mediated recruitment (mNOX-E36, a CCL2 inhibitor, or cenicriviroc, a CCR2/CCR5 dual inhibitor) and (ii) that adoptive transfer of bone marrow monocytes exacerbated tissue damage (66). Initiation of repair and control of inflammation is mediated following a phenotypic switch in hepatic macrophages toward a pro-resolution, hepatoprotective subset expressing IL-10, IL-4 and IL-13 (67–69). This maturation event is dependent on colony stimulating factor 1 (CSF1) and secretory leukocyte protease inhibitor (SLPI) in areas of hepatic necrosis (70–72). The emergence of CCR2^low^CX_3_CR1^high^ cells is also associated with the expression of vascular endothelial growth factor A (VEGF-A) which promotes repair of the vascular architecture, and increased phagocytic capacity to remove dead and dying cells (72–74). In murine models this reparative pathway can be disrupted via modulation of CCR2 signaling or depletion of macrophages through treatment with liposomal clodronate, indicating that both tissue resident and infiltrating myeloid cell populations orchestrate repair (62, 63, 75).

Similar findings have been described in patients with ALF. Clusters of CCR2^+^ macrophages are seen in patients with APAP-induced liver failure (66) and increased serum CCL2 levels are associated with an unfavorable prognosis (49). A pro-resolution population of MerTK^+^HLA-DR^high^ cells has been identified in circulatory and tissue compartments of patients with ALF (72, 76). Analysis of these macrophages determined that they secreted anti-inflammatory mediators and exhibited reduced responses to bacterial challenge, consistent with an anti-inflammatory immune tolerant function. This is supported by the fact that APAP-treated *Mer* knockout animals exhibited persistent liver injury and inflammation associated with a defect in efferocytosis (72).

The pathways involved in other acute injury settings also result in activation of KC following hepatocyte damage mediated by T-cells (concanavalin A), oxidative stress (I-R), heat (sterile injury), or virus induced apoptosis (hepatitis viruses). During viral infection of humans KC increase in number and drive the infiltration of other immune cell populations through the production of inflammatory cytokines such as IL-1β, IL-18, and TNF-α (77–80). KC expression of IL-6, IFN-γ, reactive oxygen species, FAS ligand, granzyme B and TRAIL has been shown to inhibit hepatitis C (HCV) replication, and induces apoptosis of infected hepatocytes (81, 82). Triggering of KC responses arises as a result of engulfment of hepatitis B viral particles (leading to production of IL-18 and NK cell stimulation) (83) or via TLR2 signaling and formation of the inflammasome, with concomitant secretion of IL-18 and IL-1β, in the case of HCV (84, 85). Conversely in the setting of chronic hepatitis B viral infection the immune response is impaired through release of IL-10 (86), reduced IL-12 expression (87) or T-cell exhaustion (88) mediated by TLR2 signaling on KCs, via upregulation of galectin-9 expression driving further immune cell exhaustion following engagement with Tim-3 (89), or through increased expression of the inhibitory ligand PDL1 (90). An excess of hepatitis B virus antigen can also dampen TLR responses which contribute to viral evasion of innate and adaptive immune responses (91). This is thought to occur through suppression of proinflammatory cytokines and expression of tolerogenic mediators (IL-10 in particular) reminiscent of the tolerogenic effects of LPS, although the signaling pathways mediating this effect may be distinct.

### Chronic Liver Disease and Contribution to Fibrosis

**Figure 3:**
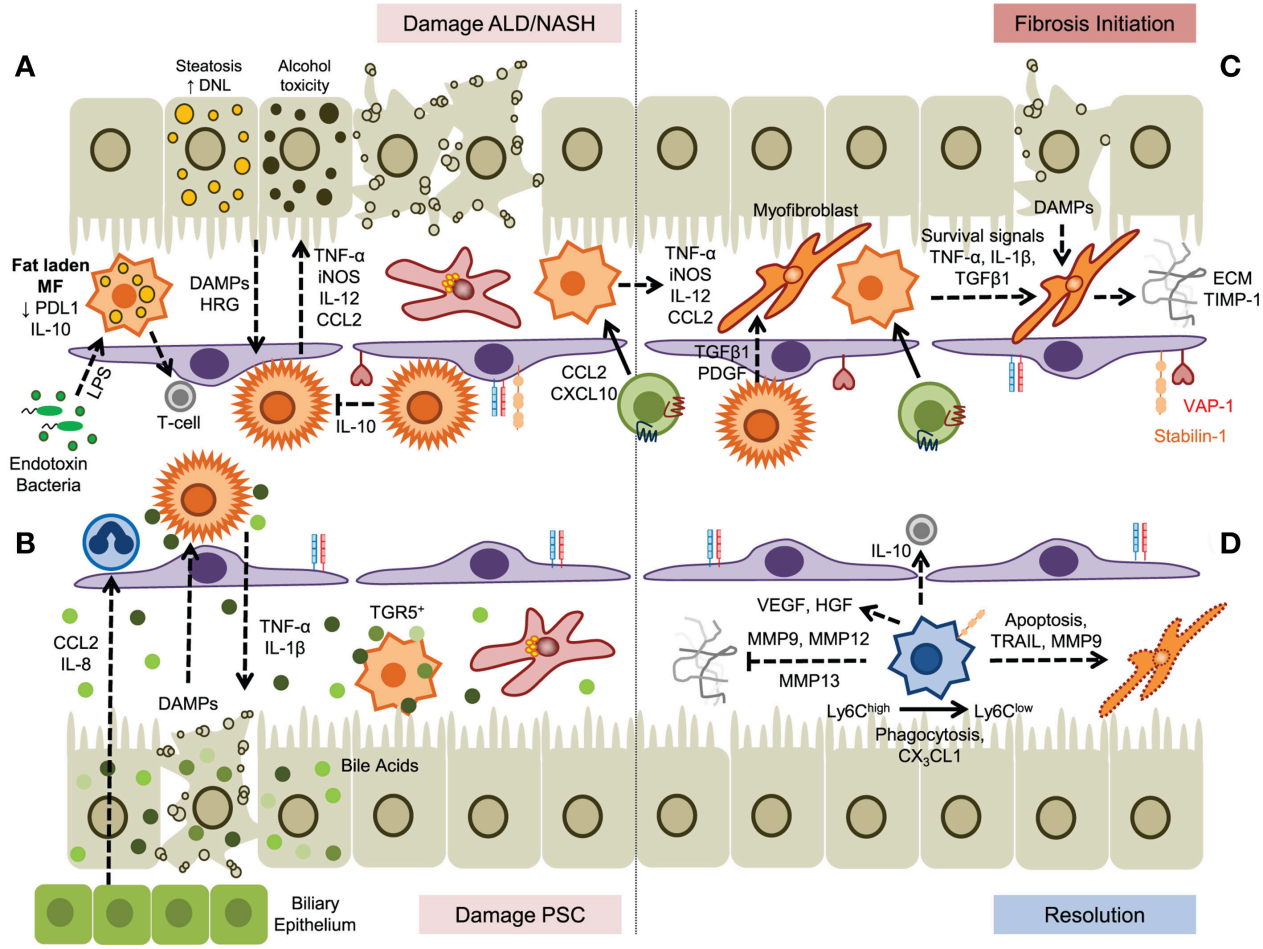
A dual role for myeloid cells in the establishment and resolution of chronic liver disease. **(A)** Hepatocyte damage driven by steatosis or alcohol toxicity activates KC which secrete proinflammatory cytokines that drive disease progression and promotes infiltration of myeloid cells. In steatotic livers fat laden macrophages exhibit impaired endotoxin responses but may prime T-cell mediated immunity. **(B)** Cholangiocyte-derived chemokines promote recruitment of hepatic neutrophils and subsequent damage to hepatocytes promotes further inflammation. Bile acids promote KC inflammasome formation; however this can be suppressed through binding of bile salts to TGR5 expressed by monocyte-derived macrophages. **(C)** Secretion of soluble factors by KC and monocyte-derived macrophages promotes fibrosis through the activation and differentiation of hepatic stellate cells, promoting survival of myofibroblasts and the generation of extracellular matrix proteins. **(D)** Resolution of fibrosis is mediated by Ly6C^low^ macrophages, generated from Ly6C^high^ precursors, by degradation of ECM by matrix metalloproteinases, induced apoptosis of hepatic stellate cells and myofibroblasts, and secretion of anti-inflammatory cytokines.

A prolonged cycle of iterative bursts of tissue damage and inflammation underlies chronic liver disease leading to fibrogenesis and ultimately in some cases cirrhosis. A proportion of patients will develop hepatocellular carcinoma on the background of continuing inflammation and fibrogenesis (92). The incidence of non-alcoholic fatty liver disease (NAFLD) and alcohol related liver disease (ARLD) has increased rapidly in recent years and following advances in the treatment of chronic viral hepatitis, attention is now switching to treating these increasingly common chronic conditions (93) (Figure 3).

NAFLD is a spectrum of disease ranging from simple steatosis (fatty liver) to non-alcoholic steatohepatitis (NASH), fibrosis and cirrhosis (with or without malignancy). The underlying pathology is driven by dysregulation of lipid metabolism and accumulation of lipid in hepatocytes. It is a systemic disease where dysregulated inflammation in adipose, and liver tissue and changes in the gut microbiome all drive the production of inflammatory mediators such as cytokines and chemokines (94). In patients with NAFLD enlarged and aggregated KC populations are seen in the liver and their presence correlates with the severity of the disease (95).

This is consistent with observations in diet-induced murine models of NAFLD where KC activation leads to triglyceride accumulation and production of proinflammatory cytokines such as TNF-α (96, 97). Murine hepatic macrophages can also receive activation signals from lipid-stimulated hepatocyte-derived extracellular vesicles via tumor necrosis factor-related apoptosis-inducing ligand receptor 2 (TRAIL-R2, also known as DR5) and receptor-interacting protein kinase 1 (98), and obese mice also show reduced expression of the glucocorticoid-induced leucine zipper (GILZ) in macrophages associated with a proinflammatory phenotype (99). In this context the development of steatohepatitis arises from chronic inflammation associated with an influx of Ly6C^+^ monocytes that enhances the proinflammatory environment through activation of liver resident cell populations (100, 101). These infiltrating monocyte subsets are recruited via chemokine receptor pairs such as CCR2-CCL2 (40, 102) and CXCR3-CXCL10 (44), or atypical adhesion molecules including vascular adhesion protein-1 and scavenger receptors (103).

Intestinal dysbiosis and hepatocyte apoptosis contribute to the inflammatory response via DAMP- and PAMP-mediated pathways respectively (104, 105) associated with increased expression and activation of receptors such as *TLR4* and *TLR9* in both humans and murine models of NASH (106). Changes in the microbiome can have complex effects on the liver altering metabolic response through the production of metabolites that enter the liver via the portal vein as well as through bacterial products such as LPS, and in the presence of a leaky gut intact bacteria are taken up by KC (107). KC also regulate anti-inflammatory responses through secretion of IL-10. In addition to its more general anti-inflammatory properties KC derived IL-10 also induces the apoptosis of proinflammatory KC allowing KC to self-regulate toward a more tolerogenic environment (108). The induction of a pro-resolution M2 KC phenotype is dependent on activation of RORα and KLF4, and provision of an activator of RORα (JC1-40) improved the symptoms of NASH in a high fat diet murine model suggesting that KC polarization is a viable therapeutic strategy (109). Immune checkpoint proteins such as Tim-3 have been detected on a range of macrophage subsets in murine models of NASH. The presence of TIM-3 limits steatohepatitis by controlling ROS induced activation of NOX2 and the NLRP3 inflammasome and secretion of IL-1β and IL-18 (110). Thus, therapeutic strategies could look at promoting the recruitment or differentiation of TIM-3 macrophages to shift the local environment toward resolution and suppression of inflammation.

Similar mechanisms of disease progression have been described for ARLD, where metabolism of alcohol in the liver drives hepatocyte cell death. In rodent models of ARLD such as the Lieber-DeCarli diet hepatic macrophages become activated to produce TNF-α, IL-6, CCL2 and ROS (111, 112) and depletion of macrophage populations with either GdCl_3_ or liposomal clodronate attenuated alcohol-induced liver inflammation (111, 113). Expression of myeloid NADPH oxidase, specifically the catalytic subunit gp91^phox^, contributes to the pathogenesis of murine ARLD by driving a switch between pro-inflammatory and pro-resolution macrophage populations. Thus, gp91^phox^-deficient animals show an increased ratio of Ly6C^high^/Ly6C^low^ intrahepatic macrophages and a diminished capacity for efferocytosis (114). Clustering of myeloid cells close to portal tracts is observed in ARLD patients (115) associated with increased levels of cytokines (IL-6, IL-8, IL-18), chemokines and macrophage activation markers that correlate with outcome and severity of disease (116–119). Gut permeability is increased in patients with ARLD leading to high levels of endotoxin in the liver resulting in a greater sensitivity of circulating monocytes from these patients to LPS (120, 121); a phenomenon also reported for resident KC isolated from alcohol-fed mice where increased sensitivity to endotoxin promoted expression of TNF-α and CCL2 (122, 123).

The role of macrophages in cholestatic diseases such as primary biliary cholangitis (PBC) and primary sclerosing cholangitis (PSC) is not well described. Accumulation of perisinusoidal hepatic macrophages in human tissue is reported in PSC but not in PBC (124) and increased infiltration of CD68^+^/CCR2^+^ cells was observed at later stages of disease in PSC including both CD206^+^ (anti-inflammatory) and iNOS^+^ (pro-inflammatory) macrophages (125). In diseases associated with cholestasis dysregulated bile acid production and excretion by cholangiocytes directly affects macrophage function and differentiation although the effects are complex. Although in mice hydrophobic bile acids have been reported to promote the formation of macrophage inflammasomes and IL-1β secretion (126, 127) other studies report activation of anti-inflammatory pathways in human macrophages by taurolithocholic acid through a PKA-mediated increase in IL-10 (128). Mice lacking the bile acid transporter *Mdr2* (*Abcb4*) develop hepatobiliary inflammation and fibrosis with some, but not all, features of PSC including an accumulation of peribiliary, proinflammatory macrophages recruited in response to cholangiocyte secretion of IL-8 and CCL2. Pharmacological treatment of mice with the CCR2/CCR5 antagonist cenicriviroc attenuated macrophage infiltration and liver injury consistent with an effector role for macrophages (125) and other rodent models have shown that the G-protein-coupled bile acid receptor, *Gpbar1* (TGR5) is expressed by macrophages to sense and respond to bile acids (129, 130). Activation of murine TGR5 leads to PKA-induced ubiquitination of NLRP3, acting as a brake on inflammasome activation (131) and dampening cytokine responses (129). In a murine model of colitis treatment with the TGR5 agonist BAR501 reduced the trafficking of Ly6C^high^ monocytes into the intestinal mucosa, reduced the expression of inflammatory genes (*Tnfa, Ifng, Il1b, Il6*, and *Ccl2*), and induced a regulatory T-cell environment through the production of IL-10 and TGF-β (132). The therapeutic potential of other TGR5 agonists such as 6α-ethyl-23(S)-methyl-cholic acid (6-EMCA, INT-777) are currently being explored in cholestatic liver disease (126, 133).

## Neutrophil Mediated Liver Injury

Neutrophils are derived from bone marrow and are released into the peripheral circulation where they play an important role in host defense and tissue healing (134), characterized by a high phagocytic capacity, the production of antimicrobial molecules and ability to shape immune responses (134, 135). The identities of neutrophil subsets and their functions are not clearly defined, with much of our knowledge arising from murine models. As a result their important roles in liver homeostasis and disease are only beginning to be understood (136, 137).

Neutrophil recruitment from the circulation into the liver is independent of selectins (138) and in many conditions is also independent of α2 integrin and ICAM-1 (139). Instead, neutrophils use CD44 to bind hyaluronan (HA) on LSEC and respond to chemokine ligands of CXCR2. A signaling network of TLR2, S100A9 and CXCL2 was shown to be necessary for neutrophil recruitment in a chronic model of liver injury in the mouse (140), while activation of TLR4 on LSEC was sufficient to induce the deposition of serum-associated hyaluronan-associated protein within the hepatic sinusoids which promoted CD44-dependent neutrophil migration in a murine model of endotoxemia (141). In sterile rodent injury models such as local thermal injury, HA-CD44 driven recruitment is less important and neutrophils use αMβ2 (Mac-1) binding to ICAM-1 (21). This pathway plays little role in septic injury because IL-10 leads to a loss of cell surface αMβ2 (142). Invading neutrophils in septic injury tend to arrest soon after infiltrating the tissue, whereas in sterile injury these cells migrate toward the focus of damage and adopt a swarming behavior which restricts neutrophil motility to within the boundary of the injury. This behavior is amplified by leukotriene B4 (LTB4) produced by the first invading neutrophils (21, 143). In sterile injury neutrophil recruitment can be promoted by ATP release from necrotic hepatocytes leading to activation of the inflammasome, and presentation of ligands for CXCR2 on the surface of the hepatic sinusoids (21). Alternatively N-formyl peptides released from dying and dead hepatocytes are detected by the formylated peptide receptor-1 on neutrophils which guide them toward the site of injury (21, 144, 145). This enables neutrophils to prioritize their responses to chemoattractant gradients that arise directly from damaged tissue over competing signals from chemokines or LTB4 and remain within the boundaries of the necrotized tissue (146–149). Live cell imaging in mice identified a non-muscle myosin II protein that was essential for neutrophil trafficking, demonstrating that myosin heavy chain 9 (Myh9) was localized in branching lamellipodia and in the uropod where it may enable fast neutrophil migration (150).

During acute liver injury, neutrophils use the receptor for advanced glycation end products (RAGE) to respond to HMGB1 released by necrotic hepatocytes (151). However, this pathway also contributes to sepsis through diminished bacterial killing by neutrophils and reduced NADPH oxidase activation (152). Neutrophils form extracellular traps by a process known as NETosis to enhance antibacterial defenses [reviewed in (153)]. Defects in NET formation have been linked to impaired efferocytosis and contribute to liver injury and sepsis in models of liver disease (154). In murine models of chronic liver disease neutrophils drive hepatocellular damage but are also associated with mechanisms of tissue repair. Myeloperoxidase secreted by neutrophils drives oxidative damage and contributes to the development of NASH in mice (155) and increased levels of myeloperoxidase activity have been detected in patients with NASH (156). The development of obesity-related inflammation in patients with NASH also correlated with an increase in the ratio of neutrophil elastase to its inhibitor α1-antitrypsin, although the ratio reduced as the disease progressed to fibrosis (157). Conversely murine neutrophils can alleviate fibrosis through secretion of MMP8 and MMP9 (158), and depletion of neutrophils improved liver function in a diet-induced model of NASH (159). Following resolution of tissue damage in sterile injury neutrophils migrate out of the tissue and back into the vasculature and, following passage through the lungs where they upregulate CXCR4, return to the bone marrow where they undergo apoptosis (160). It is not currently known if this process contributes to the pathogenesis of other hepatic diseases.

### The Contribution of Dendritic Cells to the Development of Liver Disease

In contrast to hepatic macrophages liver dendritic cells are scarce and mostly scattered in the portal region where they capture antigens delivered by via the portal vein (161). Dendritic cells can also translocate from blood to lymph via the hepatic sinusoids to concentrate in regional perihepatic lymph nodes (162). Hepatic dendritic cells comprise plasmacytoid DCs (pDCs) and classical (myeloid) DCs (cDCs) which express high levels of MHC-Class II molecules (e.g., HLA-DR) but are negative for other hematopoietic lineage markers (163). In general hepatic DCs are tolerogenic and inherently anti-inflammatory, but they can gain pro-inflammatory properties in the setting of chronic liver injury (164, 165). Plasmacytoid DCs identified as lin^−^CD11c^int^MHC-II^int^PDCA-1^+^Siglec-H^+^ in some respects resemble B-cells and represent the most abundant subset in the murine liver under steady-state conditions (163, 166). Human pDCs are characterized by BDCA-2 and CD123 expression, but occur less frequently than in mice (167). This cell population responds to TLR7/8 ligands and mediate antiviral immunity by secreting type I interferons such as IFN-α but are less potent T-cell inductors. Classical DCs comprise two subtypes: cross-presenting lin^−^CD11c^+^CD11b^−^CD103^+^CX_3_CR1^−^ DCs (mainly interacting with CD8^+^ T-cells via MHC-I) and conventional lin^−^CD11c^+^CD11b^+^CD103^−^CX_3_CR1^+^ DCs (presenting MHC-II bound antigens to CD4^+^ T-cells) which correspond to human CD141 (BDCA-3) and CD1c (BDCA-1) DCs respectively (61, 163). In human liver CD1c^+^ DCs prevail in contrast to mice (168). Another recent nomenclature differentiates hepatic DCs based on lipid content with high-lipid liver DCs inducing robust T-cell activation and cytokine secretion whereas low-lipid DC promote immune tolerance in both mice and humans (169).

Several factors contribute to the tolerogenic nature of hepatic DCs. When compared to splenic DCs, hepatic DCs were shown to be relatively immature (less CD40, CD80, CD86, CD83) with a reduced capacity to cross-present antigen to T-cells. Human DCs secrete high levels of IL-10, but less IL12p70 upon LPS-exposure (165) thereby contributing to endotoxin tolerance in the healthy liver (170). According to some reports hepatic DCs also predominantly induce regulatory and IL-4 secreting T-cells (171). During homeostasis low levels of circulating LPS trigger the expression of indoleamine-2,3-dioxygenase in human pDCs which catalyzes the production of immunoregulatory metabolites (172, 173). Following CpG stimulation murine pDC also fail to release abundant class I interferons owing to high NOD2 expression (174). Interestingly, circulating DCs that cross the hepatic sinusoids to reach the afferent lymphatics are educated by the hepatic microenvironment to adopt a regulatory phenotype, emphasizing the inherent tolerogenic phenotype of the hepatic niche (175).

Compared to other myeloid cells such as macrophages and monocytes the role of DCs in the initiation and progression of liver diseases is poorly defined. After switching from a regulatory to a proinflammatory state hepatic DCs can exacerbate acute liver injury in certain murine models (176, 177) whereas in human fatty liver disease there is emerging evidence they are protective by removing cellular debris and restricting DAMP driven activation of innate effector CD8^+^ T-cells (178). CD103^+^ DCs might be central to this response as *Batf3* deficient mice that lack CD103 displayed a more aggressive course in an experimental model of NASH (179). The failure to clear HCV has been associated with a reduced capacity of pDCs to secrete antiviral IFN-α and their ability to stimulate inhibitory T-cell receptors such as PD-1, TIM-3, and CTLA-4 (180–182). Thus, impaired DC activation in HCV infection might favor T-cell unresponsiveness leading to viral immune escape and persistence. DCs are not the only APCs within the liver. The liver's unique metabolic functions and constant exposure to gut antigens and gut-derived microbial products has resulted in a complex system for regulating immune responses in which DCs, endothelial cells and stromal cells may all contribute to presenting antigens and maintaining immune homeostasis (175).

Some studies have suggested that DCs may play a role in driving fibrogenesis beyond their ability to activate immune responses. However, the data are not compelling and most evidence points to DCs being largely dispensable for the progression of fibrosis. Although the expansion of CD11b^+^ DCs has been observed during hepatic fibrogenesis in mice (183, 184), DCs are thought to promote resolution rather than progression of fibrosis. For example depletion of DCs during the regression phase of murine liver fibrosis significantly impairs tissue repair whereas *in vivo* expansion or adoptive transfer of purified DCs enhanced fibrosis reversal. This pro-resolution effect was mediated by MMP9 activity and clearance of activated hepatic stellate cells (185). Moreover, due to their anti-angiogenic properties DCs can counteract the profibrotic effect of VEGF mainly by expressing the VEGF receptor 1 (sFLT1) thus reducing the bioavailability of VEGF (186).

### Role of Macrophages in Fibrosis Progression and Resolution

Liver fibrogenesis was previously regarded as a unidirectional process with little chance of resolution once scar tissue has formed. However, evidence now shows that even advanced fibrosis and in some circumstances cirrhosis are at least partially reversible if the cause of liver injury can be eliminated (187). This concept has been demonstrated in both experimental models of chronic liver injury (188–190) and in human liver disease (191). For example in humans successful treatment of chronic viral hepatitis can lead to a marked improvement in liver architecture indicating that the liver has the potential for regeneration and remodeling of scar tissue. A landmark study by Marcellin et al. demonstrated that following 5 years of treatment of chronic Hepatitis B infection with tenofovir disoproxil fumarate, cirrhosis could be reverted in 74% of cases (192).

Kupffer cells and infiltrating monocyte-derived macrophages are crucially involved in this process of tissue remodeling (Figure 3) and it is clear that hepatic macrophages can play context-dependent fibrogenic and fibrolytic roles due to their heterogeneity and plasticity. For example hepatocyte-derived HRG, a non-inflammasome activating factor contributing to KC stimulation, favors a profibrotic phenotype of murine hepatic macrophages. This was demonstrated in HRG-deficient knockout mice where liver fibrosis was significantly attenuated in diet and toxin-induced models of liver injury (193). Similarly in humans interleukin-34 and macrophage colony-stimulating factor (M-CSF) promote a profibrotic phenotype in hepatic macrophages in the setting of chronic viral hepatitis (194). KC neutralize circulating endotoxins during homeostasis and release anti-inflammatory mediators such as IL-10 during low-level lipopolysaccharide (LPS) exposure (26). However, dysbiosis and translocation of gut bacteria and bacterial products due to intestinal barrier dysfunction result in the excessive presence of PAMPs within the hepatic microvasculature that reach the liver via the portal vein (195) which, in murine models, drives inflammasome activation of profibrotic hepatic stellate cells (196, 197). In a recent publication the cell-specific innate immune receptor triggering receptor expressed on myeloid cells-1 (TREM-1) was reported to promote hepatic inflammation and fibrosis in mice and humans (198), and inhibition of TREM-1 in mice ameliorated inflammation and macrophage and neutrophil activation in a mouse model of ARLD (199).

Chemokines released by KCs shapes the subsequent phase of hepatic inflammation. The CXC chemokines CXCL1, CXCL2, CXCL8 attract neutrophils whereas CCL2 is the major chemokine that governs influx of bone-marrow-derived monocytes (5). Hepatic stellate cells are another important source of CCL2 and there is a bidirectional relationship between pro-fibrotic effector cells and hepatic macrophages (197) as shown by the ability of HSC to respond to CCL2 and CCL5 produced by hepatic macrophages. Bone-marrow chimeric mice were used to show that activation of CCR2 and CCR5 in HSC drives fibrogenesis through stimulation of HSC migration and collagen production, whereas CCR1 acts solely on monocytes/macrophages (200–202). In humans macrophages exposed to HCV serum synthesize CCL5 and activate hepatic stellate cell confirming the murine data (203). CCL3 deficient mice display reduced HSC proliferation and migration and attenuated fibrogenesis (204). Secretion of CCL3 was shown to be dependent on the expression of the scavenger receptor Stabilin-1 by a specific macrophage population, and genetic deletion of *Stab1* led to diminished anti-fibrotic responses in diet and toxin-induced murine models of liver disease (205).

The release of CCL2 during early hepatic injury in mice augments the intrahepatic pool of macrophages by selectively attracting bone-marrow derived inflammatory CCR2^+^CX_3_CR1^low^Ly6C^high^ monocytes but not CCR2^−^CX_3_CR1^high^Ly6C^low^ counterparts (56). The expansion of hepatic macrophages is maintained during iterative episodes of liver injury that drive fibrogenesis (56) and CCR2 directed inhibition of monocyte recruitment during liver injury in murine models reduces liver scarring (56, 200, 206, 207). Targeting CCR2 with either the small molecule inhibitor cenicriviroc (208–210) or the L-enantiomeric RNA oligonucleotide mNOX-E36 (211) achieved similar results. In line with this data from the phase 2b Centaur trial revealed that treatment with cenicriviroc reduces fibrosis in patients with NASH after 1 year of treatment (212). Despite these advances, the profibrotic role of monocytic CCR2 is not fully understood following recent studies demonstrating that CCR2 expressed by monocytes/macrophages is dispensable for liver fibrogenesis (200). This was confirmed following further studies in mice which revealed that CCR1 (which binds CCL3 and CCL4), CCR8 (which binds mainly CCL1) and CCR9 which binds CCL25 are also involved in recruiting monocytes to the site of hepatic injury during fibrogenesis (42, 201, 213).

Both monocyte-derived macrophages and Kupffer cells promote fibrogenesis by secreting TGF-β and galectin-3, which drive transdifferentiation of HSCs into matrix secreting myofibroblasts (56, 214–216). Hepatic macrophages are also implicated in the survival and activation of HSC through secretion of IL-1β and TNF-α [in a NF-κB activation-dependent fashion (183)] or via IL-4 and IL-13 secretion in Th2-dominated rodent injury models such as parasitic infections (217). Oncostatin M (OSM) might also function as a potent regulator of hepatic macrophage/HSC interaction by enhancing the expression of profibrotic and mitogenic genes such as TGF-β and PDGF in bone-marrow derived infiltrating macrophages, with macrophage-depleted livers being largely protected from OSM-induced fibrosis (218). Interestingly, the profibrotic effect of KC-secreted TGF-β is retained following inhibition of CCL2-dependent monocyte in experimental steatohepatitis (102) which might impede the effectiveness of CCL2/CCR2 based therapies to treat liver fibrogenesis.

The first reports of hepatic macrophages driving fibrosis resolution were published alongside data describing the profibrogenic nature of Kupffer cells and monocyte-derived macrophages in liver injury. This apparent paradox was clarified when Duffield and colleagues reported a dual role for macrophages during different phases of chronic liver injury in mice. They used a CCl_4_ model to show that mice in which hepatic macrophages were selectively depleted exhibited less matrix deposition at advanced stages of fibrogenesis but more fibrosis when macrophages were depleted during the resolution phase (219). These data suggested the existence of distinct macrophage populations within the liver that fulfill opposing functions according to the disease stage. A subsequent study by the same group confirmed this by reporting the accumulation of macrophages around scar fibers during the resolution phase that were capable of degrading ECM through expression of matrix metalloproteinase protein 13 (MMP13) (220). These scar-associated macrophages are also equipped with MMP9, MMP12 and TRAIL, and contribute to the disruption of scar tissue and induction of fibroblast apoptosis (187). However, these studies did not identify which hepatic macrophage population gave rise to this profibrolytic subset. One of the first lines of evidence that monocyte-derived macrophages might be responsible stems from a paper showing that CCR2 deficiency is protective during fibrogenesis but hinders scar removal during the regression phase following cessation of CCl_4_ challenge in rodents. The putative mechanism is a balance between levels of tissue inhibitor of metalloproteinase-1 (TIMP1) and MMP1 and MMP13 mRNA, in the liver (207). This study also determined that profibrotic and pro-resolution macrophages share the same precursor cells; a hypothesis supported by a seminal paper in 2012 showing that in mice Ly6C^high^ inflammatory macrophages undergo a phenotypic switch to an anti-inflammatory and anti-fibrotic “restorative” CD11b^high^F4/80^int^Ly6C^low^ subtype (52). The accumulation of Ly6C^low^ macrophages producing matrilytic MMPs peaked at the maximum point of fibrosis resolution whereas the early phase of liver parenchyma damage is dominated by freshly recruited inflammatory CCR2^+^Ly6C^high^ macrophages. Phagocytosis of cell debris drives macrophage transdifferentiation toward a restorative Ly6C^low^ phenotype (52). The concept of a context dependent hepatic macrophage plasticity was demonstrated using sterile liver inflammation models in which CCR2^high^CX_3_CR1^low^ macrophages accumulate early after focal tissue injury in a ring-like structure and then give rise to a reparative CCR2^low^CX_3_CR1^high^ phenotype which facilitate wound repair (14). There is also evidence that *in situ* reprogramming of infiltrating macrophages from a profibrotic to an antifibrotic subset is controlled by the CX_3_CR1/CX_3_CL1 axis, which promotes macrophage survival and imprints an anti-inflammatory state. Consequently, CX_3_CR1 knockout mice display enhanced tissue damage and fibrosis after bile duct ligation and CCl_4_ exposure (221, 222). Though circulating Gr1^low^ (Ly6C^low^) CX_3_CR1^+^ monocytes show patrolling behavior in blood stream (34) there is no data so far to support the idea that these cells are directly recruited to the inflamed liver and thereby perpetuate fibrosis resolution. Never the less, this cannot be excluded since extravasation of CX_3_CR1^+^ monocytes into affected organs has been demonstrated in models of myocardial infarction and lung injury (223, 224).

Translating findings from rodent models into patients is not straightforward. Most importantly—as outlined above—human liver macrophage subsets lack well-defined surface marker patterns that allow for distinction of resident Kupffer cells from infiltrating monocytes. For example CD68^+^ which is deemed to be a macrophage marker in mice can also be detected on circulating monocytes in human, and although gene profiles show overlap between murine Ly6C^high^ and “classical” human CD14^++^CD16^−^ monocytes and murine Ly6C^low^ and “non-classical” human CD14^+^CD16^++^ monocytes (31) there are clear functional differences. In addition, it is difficult to integrate the “intermediate” CD14^++^CD16^+^ subset into the murine nomenclature (225). In general, CD16^+^ monocytes are enriched in the liver in comparison to peripheral blood even under steady state conditions (46), with increased numbers being observed in patients with cirrhosis (47). CD14^+^CD16^−^ cells can acquire CD16 expression under the influence of soluble factors present in the diseased liver such as IL-10 and TGF-β, and CD16^+^ monocytes display higher phagocytic capacity and can secrete both pro- and anti-inflammatory cytokines upon LPS stimulation thus resembling both Ly6C^low^ and Ly6C^high^ monocytes/macrophages in mice. Of note, CD16^+^ but not CD16^−^ monocytes can directly activate human HSC (47). Rodent models also lack the highly-crosslinked scar tissue observed for patients with advanced fibrosis, and as a result macrophage-mediated resolution of fibrosis is accelerated in murine models compared to humans. Therefore, further research is warranted to better define disease-specific characteristics of hepatic monocyte and macrophage subsets in human diseases. Despite these differences there are some striking parallels between mice and humans. For example, the CCL2/CCR2 axis plays a similar role in fibrosis and macrophage infiltration to the inflamed liver in mice and humans, and TREM-1 is emerging as an additional shared marker.

## Malignancy

**Figure 4:**
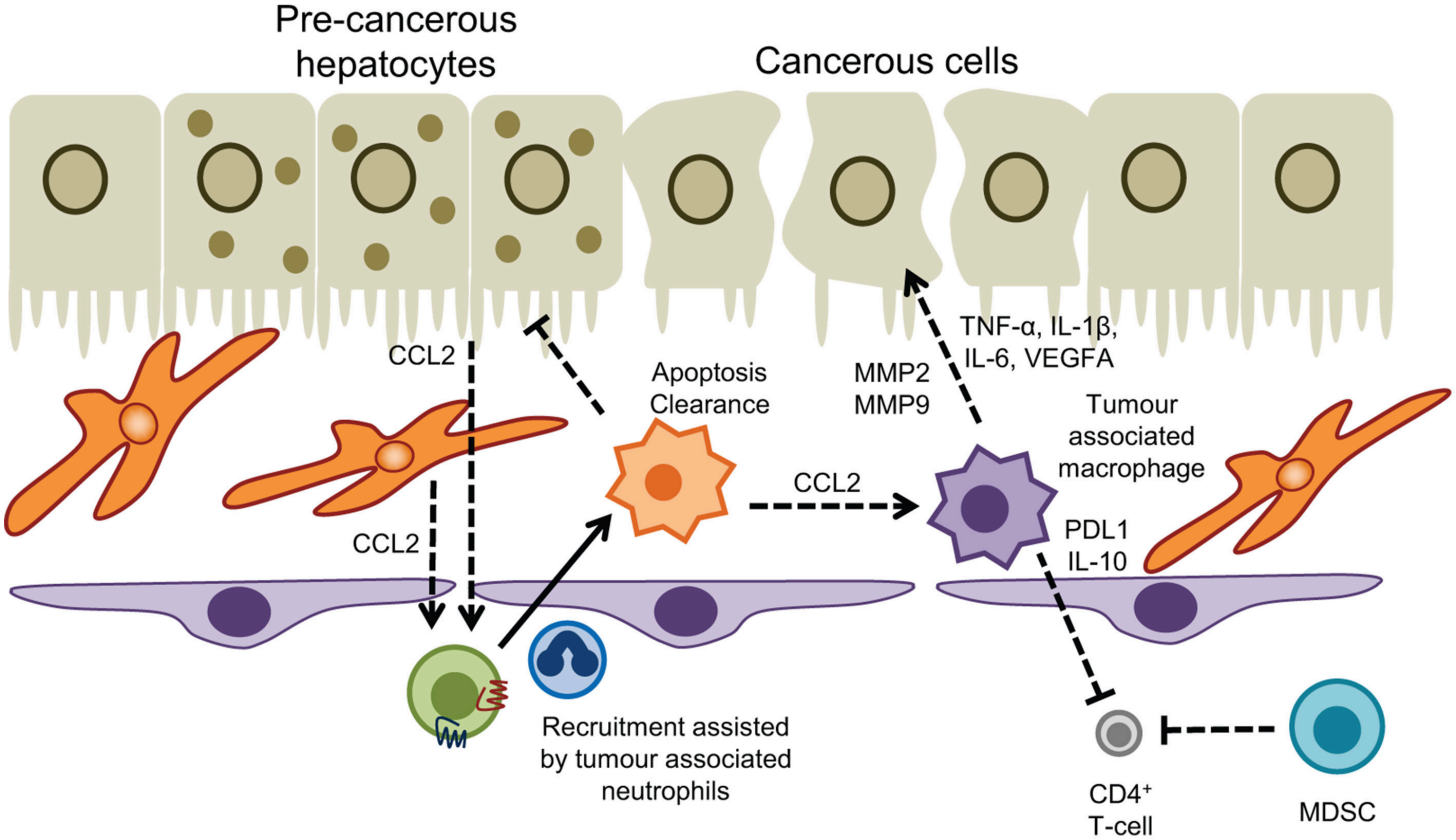
Myeloid cells in hepatic malignancy. Tumor associated macrophages promote cancer cell proliferation and neoangiogenesis, and act in concert with myeloid-derived suppressor cells to dampen T-cell immunosurveillance. Conversely recruitment of monocytes into the tumor microenvironment driven by CCL2 produced by hepatocytes and hepatic stellate cells, and interactions with tumor associated neutrophils promotes apoptosis and clearance of pre-cancerous hepatocytes to prevent HCC.

Myeloid cell subsets are linked to virtually all steps in the natural course of tumor formation and spreading in the liver. Extensive research has shown a prominent role for tumor associated macrophages (TAM) and MDSC in the development of hepatocellular carcinoma (HCC), an archetypical inflammatory tumor in which chronic inflammation drives liver cancer pathogenesis, invasion, and metastasis (226). In the context of liver carcinogenesis monocytes/macrophages exert dualistic functions in a stage-dependent manner with CCR2^+^ CCL2-responsive monocytes promoting tumor surveillance through the elimination of senescent premalignant hepatocytes in healthy livers (Figure 4), whereas in established tumors monocytes/macrophages are reprogrammed to silence NK cells resulting in tumor growth (227, 228). Tumor associated macrophages derived from infiltrating monocytes are a dominant cellular component of human tumor stroma with increased density of TAMs in peritumoral margins being closely linked to poor prognosis (229, 230). Consistent with this the absence of macrophages correlated with improved patient survival (231). In both humans and murine models TAMs drive tumorigenesis by sustaining inflammatory pathways mediated through secretion of cytokines such as TNF-α and IL-6 that suppress hepatocyte apoptosis and induce proliferation in response to tissue damage (232–234). The surface receptor TREM-1 expressed on myeloid cells governs the secretion of proinflammatory mediators and engagement of TREM-1 in hepatic macrophages has been shown to trigger carcinogenesis (235). *Trem1*-deficient mice treated with diethylnitrosamine (DEN) were protected from malignancy due to attenuation of cytokine secretion (e.g., IL-6, IL-1β, TNF, CCL2) and ablation of inflammatory signaling pathways (p38, ERK1/2, JNK, MAPK, and NF-κB) in KC (235).

The inflammatory environment present in chronic liver injury facilitates the recruitment and retention of monocyte-derived TAMs which promote tumorigenesis in a MMP2/MMP9 dependent fashion. This was demonstrated by comparing the seeding of injected HCC cells in healthy livers with CCl_4_ preconditioned livers where an alternatively-activated macrophage population (M2-like) were enriched in the tumor environment (236). Infiltrating TAMs are frequently reported to resemble alternatively-activated macrophages (237–239) although the dichotomous approach of M1/M2 polarization does not fully reflect the entire spectrum and heterogeneity of tissue macrophages. Nevertheless, reversal of M2-like polarization in experimental HCC has yielded promising results in containing tumor progression (240) with TIM-3 and Wnt ligands identified as critical drivers of alternative activation of TAMs and HCC growth (239, 241). In patients, total immune cell infiltration into HCC correlated with M1-like macrophage populations and a more favorable prognosis (242).

The CCL2/CCR2 axis is a promising novel target in HCC therapy. Antagonism of CCR2 by the compound RDC018 not only reduced TAM infiltration but also restored anti-tumor immune response and ameliorated HCC outcome in murine models of HCC (243). Tumor associated neutrophils (TAN) provide an important source of CCL2 in HCC and can act synergistically with TAM to support liver tumor progression (244). In humans neutrophil extracellular traps can also promote inflammation and development of HCC on the background of NASH, driven by the presence of free fatty acids (245); however our understanding of the precise role played by neutrophils in liver cancer remains elusive. One striking feature of TAM is the induction of an immune suppressive microenvironment that disrupts anti-tumor immunity. For example, release of regulatory cytokines such as TGF-β and IL-10 by TAM impair Th1 and cytotoxic T-cells but promote regulatory T cells and Th2 activity all of which facilitate tumor growth. TAMs also express high levels of PDL1, galectin-9, and indoleamine-pyrrole 2,3-dioxygenase (IDO) that foster T-cell exhaustion and prevent effective anti-tumor immune response (241). In HCC the expression of PDL1 by TAMs correlated with increased tumor burden and the intensity of the protein was associated with high mortality and reduced survival (246). MDSC share many mechanisms with TAM to protect from HCC-targeted T-cell activity, and the net effect of MDSCs in HCC nodules and peritumoral stroma is progression of the tumor (241). Furthermore, MDSC reduce the tissue availability of arginine and cysteine, which are essential for T-cell proliferation and impede NK cell cytotoxicity and development via NKp30 receptor (241).

## Rational Design of Therapeutic Strategies Targeting Myeloid Populations

**Figure 5:**
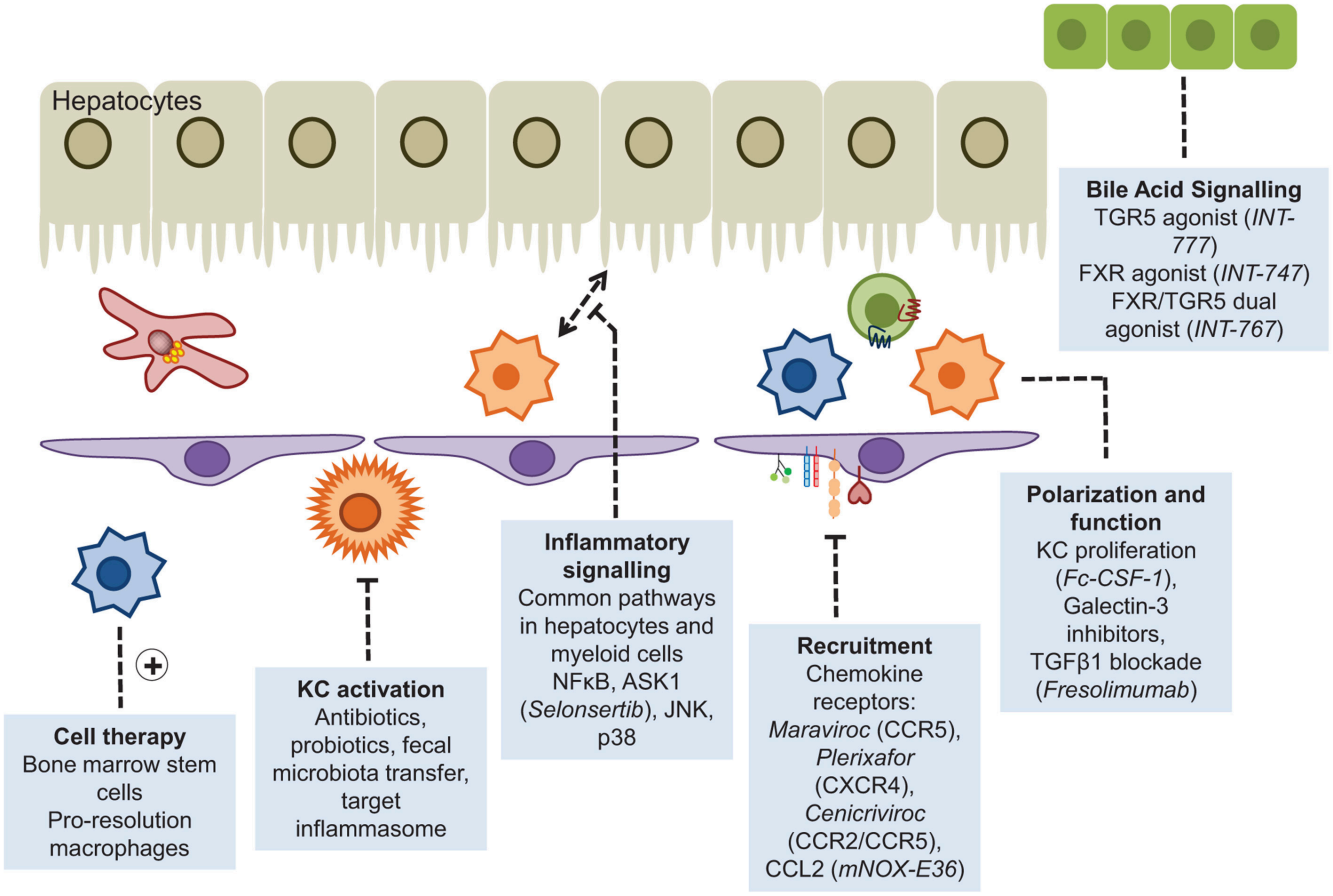
Myeloid cells as therapeutic targets. Approaches that have been adopted to enhance or diminish the role of myeloid cells in liver disease include disruption of the recruitment cascade or inflammatory signaling pathways, and augmented pro-resolution responses through cellular infusions of stem cells or the provision of agonists driving macrophage polarization.

There is a major unmet need for effective therapies to prevent or reverse liver fibrosis particularly in the context of a major increase in fatty liver disease and the continuing high prevalence of alcoholic cirrhosis (247). Macrophages have the dual potential to serve as therapeutic targets and as treatment vehicles for inflammation-induced liver fibrosis and carcinogenesis (248). In principle, macrophages can be targeted at different stages of disease and different subsets of monocyte macrophages can be targeted. Such strategies include (i) attenuation of Kupffer cell activation by anti-inflammatory compounds; (ii) inhibition of macrophage precursor cell (i.e., monocyte) recruitment to the injured liver; (iii) manipulation of macrophage polarization and differentiation to facilitate transition toward a restorative reparative phenotype (iv) infusion of beneficial pro-restorative macrophages (248) (Figure 5). Interference with chemokine pathways to restrict influx of inflammatory monocytes is one of the most advanced approaches. As stated earlier, the CCR2/CCR5 antagonist Cenicriviroc has entered phase 2b clinical trials with promising results reported after 12 months treatment of NASH-related fibrosis (212). The current options in targeting macrophages in the context of liver disease have recently been comprehensively summarized (248). Adoptive cell therapy using hematopoietic stem cells or macrophages is an approach that is attracting increasing interest. The first studies reporting efficacy of bone marrow cell transfer in murine models of liver fibrosis were published almost 15 years ago when injection of bone marrow cells was shown to cause MMP9-dependent reduction in ECM deposition in response to CCl_4_ (249). In a study by Thomas et al. in 2011 bone-marrow derived macrophages (BMM) were prepared *in vitro* by stimulation with CSF-1 and subsequently injected into the portal vein of mice with long-term CCl_4_ induced fibrosis. The infused cells did not conform to the M1/M2 paradigm but expressed IL-10, TWEAK, and MMP13, which are known to suppress inflammation and to promote cell regeneration and fibrolysis. Treatment significantly reduced liver scarring by promoting myofibroblasts apoptosis, MMP-induced degradation of ECM and by stimulating liver regeneration. In contradistinction, non-purified whole bone marrow cells increased liver fiber content (250). Similar results were obtained in another study showing that IL-10 producing CD11b^+^Gr1^+^ myeloid cells account for the tissue remodeling effect of bone marrow transplantation in liver fibrosis (251). BMM also ameliorate oxidative stress and reduce production of the potent profibrotic cytokine IL-13 (252). Interestingly, macrophages derived from pluripotent embryonic stem cells exhibit comparable antifibrotic effects to BMM though these cells tend to resemble resident Kupffer cells rather than infiltrating macrophages (253). A contributing factor to the success of bone marrow derived macrophage transplantation in liver fibrosis could be activation of the sphingosine-1-phosphate receptor (S1PR) that critically controls BMM motility (254). Mice treated with FTY720 which triggers S1PR internalization retained infused c-kit^+^/sca1^+^/lin^−^ hematopoietic stem cells in the liver due to a failure of the cells to egress into the draining lymph. This was associated with reduced scarring in methionine-choline-deficient diet fed and CCl_4_ treated mice (255). Further studies are needed to dissect whether S1P/S1PR antagonism also augments the antifibrotic effects of transplanted BMM. Despite the promising experimental findings human cell therapy trials in advanced clinical cirrhosis have so far proven disappointing. The REALISTIC trial tested the efficacy of G-CSF mobilized and autologous infusions of CD133^+^ stem cell therapy in cirrhosis but failed to show any improvement in liver function with more complications in the treatment group (256). This is perhaps unsurprising given that resolution will only occur if the right cells are infused into the right microenvironment at the right disease stage. This requires the design of more sophisticated precision medicine trials. Such studies are underway (257, 258) but we are only at the start of understanding macrophage therapy for liver diseases.

## Author Contributions

CW, HZ wrote parts of the manuscript. DA edited and finalized the manuscript.

### Conflict of Interest Statement

The authors declare that the research was conducted in the absence of any commercial or financial relationships that could be construed as a potential conflict of interest.
